# An Effective and Interpretable EEG-Based Depression Recognition Method Using Hybrid Feature Selection

**DOI:** 10.3390/bioengineering13040410

**Published:** 2026-03-31

**Authors:** Xin Xu, Qiuyun Fan, Shanjing Ju, Ruoyu Du

**Affiliations:** School of Communication and Information Engineering, Nanjing University of Posts and Telecommunications, Nanjing 210003, China; xuxin@njupt.edu.cn (X.X.); 1223014112@njupt.edu.cn (Q.F.); b23100225@njupt.edu.cn (S.J.)

**Keywords:** depression, electroencephalography (EEG), feature extraction, feature selection, machine learning

## Abstract

Recent studies on EEG-based automated depression detection have primarily depended on complex deep learning models. While these methods improve classification performance, their practical application is limited by high computational complexity, challenging training processes, and poor interpretability. This paper proposes an efficient method for depression recognition, which extracts multi-domain features from preprocessed EEG signals and selects the most discriminative feature subset by integrating the rapid preliminary screening capability of RankSearch with the interactive optimization ability of the Genetic Algorithm (GA). Our approach first eliminates redundant features efficiently through RankSearch, then deeply explores inter-feature relationships via GA, significantly enhancing classification performance while maintaining feature-level interpretability. Using the optimized feature subset, we evaluate performance with multiple machine learning classifiers (Decision Tree, KNN, Random Forest, SVM, XGBoost). Experiments on the public HUSM dataset demonstrate superior performance under rigorous cross-validation (accuracy = 95.08%, sensitivity = 95.99%, specificity = 94.30%, F1-score = 95%, AUC = 0.9514), with feature importance analysis further confirming interpretability. Compared to existing models, our method achieves lower computational complexity and higher clinical practicality, offering a more efficient technical solution for objective depression diagnosis.

## 1. Introduction

According to data from the World Health Organization (WHO), depression affects over 264 million people worldwide and is associated with approximately 35.8% of suicide cases [1]. Annually, about 30–35% of patients with Major Depressive Disorder (MDD) attempt suicide, with 2–15% ultimately losing their lives to depression [2]. Traditional diagnostic methods primarily rely on clinicians’ subjective evaluations and patients’ self-reports, which suffer from limitations such as low diagnostic accuracy and susceptibility to bias. In recent years, resting-state electroencephalography (rsEEG) has emerged as a widely used neuroimaging technique in mental health research due to its non-invasive nature, high temporal resolution, and ease of acquisition [3].

Conventional machine learning approaches rely heavily on manually engineered features, with their performance being critically dependent on feature representativeness. Akbari et al. employed EEG phase space reconstruction and geometric features to train SVM/KNN classifiers [4], yet their feature selection process required domain expertise and showed limited generalizability. Sun et al. discovered significant correlations between depressive symptoms and long-range connectivity strength between the left frontal and right parieto-occipital lobes in MDD patients using EEG signals and novel functional brain network analysis [5], suggesting potential biomarkers. Pizzagalli et al. demonstrated that left prefrontal cortex activation levels might reflect individual sensitivity to positive stimuli, with hypoactivation potentially leading to reward processing deficits that induce or maintain depressive symptoms [6]. Liu et al. compared resting-state EEG between first-episode depression patients and healthy controls, finding that features like β and γ band power showed superior classification performance during eyes-open states [7]. While traditional machine learning methods demonstrate stable performance in specific scenarios, their reliance on manual feature engineering has driven researchers toward more adaptive deep learning techniques. For instance, Ying et al. proposed FCAN, a lightweight attention-based model using functional connectivity that leverages EEG signals and their coherence matrices for depression detection, highlighting the crucial role of prefrontal functional connections [8]. Sam et al. developed a novel approach combining long short-term memory networks with spiking neural networks (SNNs), where biologically plausible SNNs simulate brain activity through spike-timing-dependent plasticity (STDP) for feature extraction, followed by LSTM classification, significantly outperforming existing deep learning methods [9]. However, their substantial computational demands and diminishing performance returns have renewed research interest in optimizing feature selection methodologies.

Conventional filter or wrapper-based dimensionality reduction methods often prove ineffective in eliminating irrelevant features. Shen et al. proposed a kernel target alignment-based optimized channel selection method that measures the similarity between the channel-selected kernel matrix and the target matrix using modified kernel target alignment, significantly improving classification performance while reducing computational complexity [10]. Cai et al. demonstrated that directly using all linear and nonlinear features could lead to dimensionality exceeding several dozen, necessitating feature selection algorithms to remove redundancy [11]. Zhu et al. developed an ALO-MARL feature selection algorithm that combines ant lion optimization (ALO) for global search with multi-agent reinforcement learning (MARL) for local feature interaction optimization, substantially enhancing both classification accuracy and feature interpretability [12]. Erguzel et al. revealed that standard ant colony optimization (ACO) in feature selection might become trapped in local solutions due to stagnation behavior, exposing the limitations of traditional algorithms in global optimization capability [13]. Most existing MDD detection methods employ single-stage feature selection approaches that tend to identify locally optimal features from high-dimensional feature spaces, failing to fully exploit synergistic effects among features. Therefore, there is an urgent need to explore novel feature selection and extraction strategies to overcome these limitations and improve both the accuracy and practical utility of depression identification.

In this work, we present an efficient and interpretable machine learning framework for depression detection. The key contributions of this study include:

- A novel two-stage feature selection strategy combining RankSearch with Genetic Algorithm (GA), which dynamically adjusts feature weights and GA’s crossover/mutation probabilities to optimize feature subsets and enhance model performance.
- Focused prefrontal electrode optimization analysis employing multi-domain feature extraction, with comprehensive evaluation of their effectiveness in depression identification.
- Systematic comparison of various machine learning classifiers with different feature selection methods, providing new perspectives and methodologies for early depression diagnosis.

## 2. Materials and Methods

**Figure 1:**
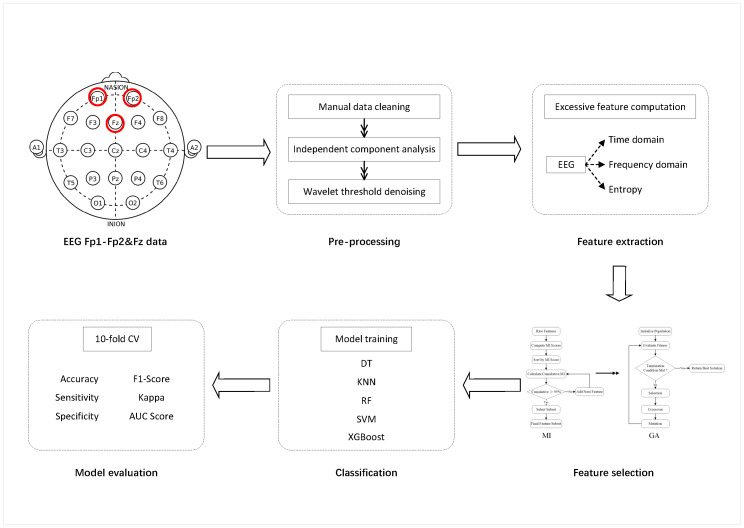
The flow of depression recognition.

The proposed depression classification and diagnosis approach comprises six main steps: EEG data collection, data preprocessing, feature extraction, feature selection, classification, and performance evaluation. Figure 1 shows the schematic flowchart of the proposed depression classification and identification method. Detailed descriptions of each step are provided below.

### 2.1. Dataset

The EEG data in this study were provided by Hospital Universiti Sains Malaysia (HUSM) for signal analysis. Following an experimental protocol approved by the HUSM Human Ethics Committee, we recruited 34 outpatient MDD patients (17 male/17 female, mean age 40.3 ± 12.9 years) and 30 age-matched healthy controls (21 male/9 female, mean age 38.3 ± 15.6 years). All participants provided written informed consent after full explanation of the study procedures. MDD patients met internationally recognized diagnostic criteria (DSM-IV) [14]. The gender imbalance in healthy controls resulted from data acquisition constraints, which we addressed through stratified cross-validation to minimize potential bias.

The dataset includes both resting-state and task-based EEG recordings. The resting-state experiment consisted of two conditions: eyes-closed (EC) and eyes-open (EO), each lasting 5 min. In this study, only the 5 min resting-state EEG signals under the eyes-closed condition were used for analysis. EEG signals were recorded using 19 channels based on the international 10–20 system for each participant.

### 2.2. Preprocessing

Raw data were preprocessed using EEGLAB toolbox in MATLAB R2019b. First, channel localization was performed according to the international 10–20 system, focusing on three electrode channels (Fp1, Fz, Fp2). The data were then bandpass filtered at 0.1–45 Hz. Subsequently, artifact-contaminated segments were removed using EEGLAB’s Clean Rawdata plugin. Independent component analysis (ICA) was applied to identify and reject noise and artifact components [15]. Prior to feature extraction, wavelet threshold denoising was performed on the raw data [16].

**Figure 2:**
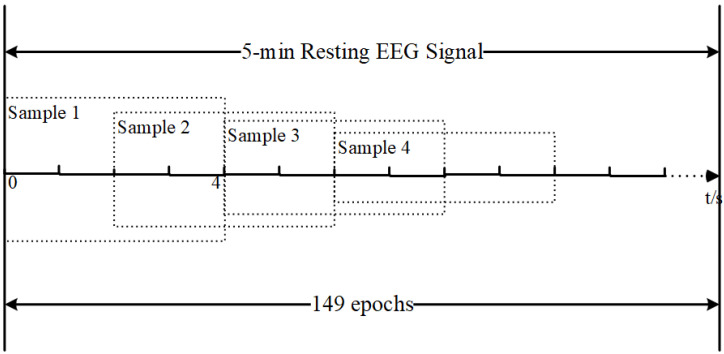
Sliding window segmentation of resting-state EEG signals.

After preprocessing, the continuous EEG signals were segmented into fixed-length epochs using a sliding window approach. As shown in Figure 2, a window size of 4 s and a step size of 2 s were adopted, resulting in overlapping EEG segments. Given a recording duration of 5 min per subject, this segmentation produced approximately 149 epochs per subject. Across all participants, this yielded approximately 9536 epochs for subsequent feature extraction and classification analysis. To ensure a fair evaluation and avoid data leakage, a subject-level cross-validation strategy was adopted. Specifically, all epochs derived from the same subject were assigned exclusively to either the training or testing set during model evaluation.

### 2.3. Feature Extraction

In Table 1, we summarize all features extracted from resting-state EEG signals. For each 4 s epoch, we extracted 6 time-domain [17], 12 frequency-domain [18], and 13 entropy/complexity features [19] from prefrontal channels (Fp1, Fz, Fp2), creating a 93-dimensional feature space (31 features × 3 channels) for brain state analysis. This multi-domain approach captures EEG characteristics across temporal, spectral, and nonlinear domains.

### 2.4. Feature Selection

This study developed a two-stage feature selection strategy. The approach sequentially combines score-ranking-based correlation analysis with search-driven global optimization to identify the most discriminative feature subset for depression recognition from the original feature set.

First, mutual information (MI) was employed as the feature importance metric to evaluate the statistical dependence between each feature and the class labels. MI demonstrates robust performance in high-dimensional EEG analysis since it captures both linear and nonlinear relationships [20]. The mathematical definition is given by:

(1) $$I\left(X;Y\right)=\sum_{x\in X}\sum_{y\in Y}p\left(x,y\right)\log\frac{p\left(x,y\right)}{p\left(x\right)p\left(y\right)}$$

where p(x,y) denotes the joint probability distribution between feature *X* and label *Y*, and p(x), p(y) represent the marginal distributions. By computing the mutual information score between each feature and the class labels, we can quantify the feature’s informational contribution to the classification task.

Subsequently, all features were sorted in descending order based on their mutual information (MI) scores, and the top-ranked features were selected to form a candidate feature subset. In this study, this stage served not only as a preprocessing step for dimensionality reduction but also provided a more compact search space for subsequent global optimization.

**Figure 3:**
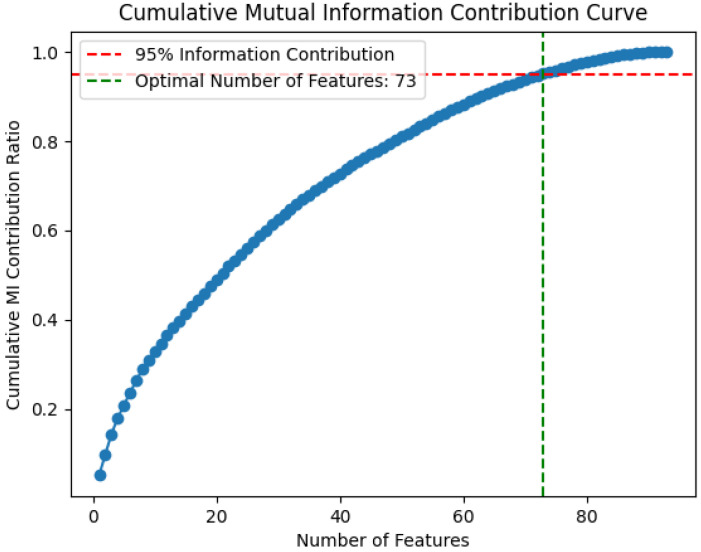
Cumulative Mutual Information vs. Feature Quantity Curve.

As shown in Figure 3, the cumulative contribution curve of features based on mutual information (MI) is presented. With a cumulative contribution threshold set at 95%, this experiment retained 73 features that collectively accounted for 95% of the total MI score. These features were preserved for subsequent feature selection steps. This strategy not only significantly reduced feature space dimensionality but also ensured the retained features contained the majority of discriminative information about the target variable, thereby enhancing both efficiency and accuracy in downstream optimization.

Building upon the candidate feature subset selected by RankSearch, we further introduced a genetic algorithm (GA) to optimize the feature subset composition. This method simulates Darwinian natural selection to perform heuristic searches in the feature space, effectively capturing high-order feature interactions and synergistic effects. By doing so, it overcomes the limitation of univariate methods that ignore interdependencies among features, The parameters of GA are shown in Table 2.

The hyperparameters of the Genetic Algorithm were determined based on commonly adopted configurations in evolutionary computation and GA-based feature selection literature. In general, crossover probabilities in the range of 0.6–0.9 and relatively low mutation rates are recommended to balance global exploration and local exploitation [21,22]. In this study, a population size of 50, crossover probability of 0.8, and mutation probability of 0.05 were selected, which fall within these widely accepted ranges. Preliminary experiments further confirmed that this configuration provides stable performance while maintaining reasonable computational cost.

The GA stage fully leverages synergistic information between features, effectively mitigating the local optimum problem in feature selection [23]. In this study, the GA not only enhanced the final classifier’s performance but also improved the compactness and interpretability of the selected feature set.

The proposed framework uses RankSearch not only as a filter but also to guide GA initialization and genetic operations. This ranking-guided search strategy improves convergence behavior, reduces randomness in feature subset exploration, and ensures that selected features are stable and informative for EEG-based depression classification. Subject-level cross-validation is applied to enhance robustness, and feature subset stability is evaluated across folds to ensure interpretability and reproducibility.

**Figure 4:**
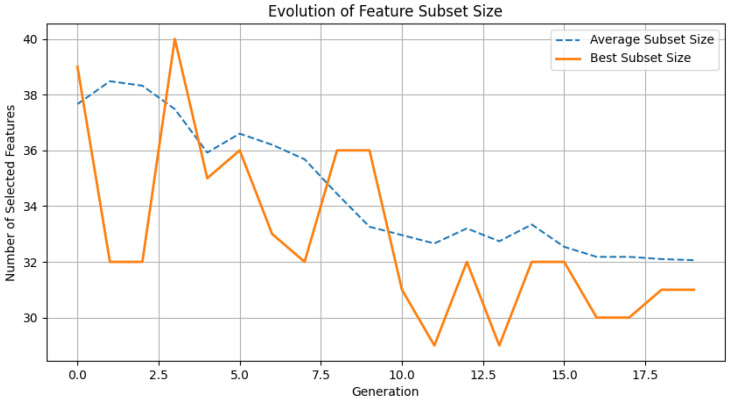
Evolutionary Trend of Feature Subset Size in Genetic Algorithm.

In Figure 4, we show the evolutionary trajectory of feature subset size during genetic algorithm (GA) optimization. The optimal subset size converges from 40 to 30 features, with population averages following a synchronous decline, demonstrating effective redundancy reduction. Stabilization occurs by generation 12, indicating balanced performance–parsimony tradeoff. This confirms GA’s ability to maintain discriminative power while enhancing interpretability and efficiency.

### 2.5. Classification Model

Following feature selection, we employed multiple machine learning classifiers for model training and evaluation using the selected depression diagnostic features, including Decision Tree (DT), k-Nearest Neighbors (KNN), Random Forest (RF), Support Vector Machine (SVM), and XGBoost. Each classifier possesses unique advantages for uncovering different aspects of the relationship between features and depression diagnosis [24].

To optimize classifier performance, we conducted hyperparameter tuning for each model using grid search. This process identified the optimal parameter configurations, ensuring peak classification performance for depression diagnosis tasks [25].

### 2.6. Evaluation Methods

To comprehensively evaluate the performance of the proposed depression diagnosis model, this study employed six key quantitative metrics: classification accuracy, sensitivity, specificity, F1, Cohen’s Kappa (κ), and AUC score. These metrics collectively assess the model’s diagnostic effectiveness from multiple perspectives:

(2) $$Accuracy=\frac{TP+TN}{TP+FP+FN+TN}$$

(3) $$Sensitivity=\frac{TP}{TP+FN}$$

(4) $$Specificity=\frac{TN}{TN+FP}$$

(5) $$F1=\frac{2TP}{2TP+FP+FN}$$

(6) $$\kappa =\frac{p_{o}-p_{e}}{1-p_{e}}$$

(7) $$AUC=\int_{0}^{1}ROC_{curve\left(\theta \right)d\theta }$$

where TP (True Positive) refers to the correctly predicted positive cases, TN (True Negative) refers to the correctly predicted negative cases, FP (False Positive) refers to the incorrectly predicted positive cases, and FN (False Negative) refers to the incorrectly predicted negative cases. po represents Accuracy, and pe is the sum of the product of actual quantities and predicted quantities divided by the square of the total sample size. The ROC curve characterizes the true positive rate (TPR = TP/(TP + FN)) at a given false positive rate (FPR = FP/(FP + TN)) threshold θ. All statistical metrics were evaluated using 10-fold cross-validation performed at the subject level, ensuring that all epochs from the same subject were exclusively assigned to either the training or testing set.

## 3. Results

### 3.1. Classification Results

In Table 3, we present the performance evaluation of the optimal feature subset across five classifiers using rigorous cross-validation. The results demonstrate the effectiveness of our two-stage feature selection, with XGBoost showing the best overall performance while KNN and SVM exhibit strengths in specific metrics. These findings are consistent with established depression diagnosis research, confirming our method’s reliability.

In Table 4, we compare classification performance using different feature sets to demonstrate the impact of feature engineering and selection. As expected, models achieved superior performance with the complete set of 93 original features, confirming the expressiveness of our comprehensive feature construction.

We systematically evaluated classification performance across different feature subset sizes. With 73 features selected by RankSearch, all metrics showed modest improvement, indicating that appropriate feature reduction enhances discriminative power by removing redundancy. The 30-feature subset obtained through our two-stage selection maintained comparable performance to the full feature set. However, further reduction to 10 features led to significant performance degradation, confirming the critical role of optimal feature selection. These results demonstrate that our method effectively identifies the most discriminative features, where moderate reduction improves model performance, while excessive reduction compromises classification accuracy.

To further evaluate the practical applicability of the proposed method in clinical scenarios, we additionally analyzed the impact of EEG recording duration on classification performance. Specifically, the data length was progressively increased from 1 to 5 min, and the classification performance of multiple classifiers was evaluated.

**Figure 5:**
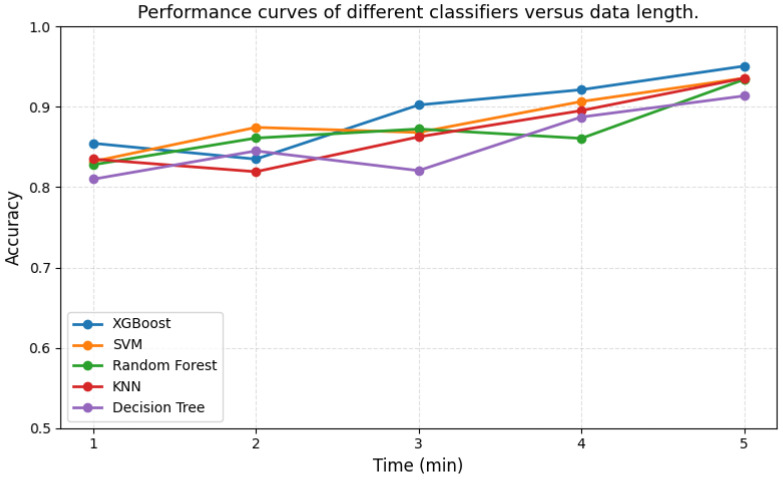
Performance curves of different classifiers versus data length.

As shown in Figure 5, classification accuracy generally improves as the recording duration increases. Notably, relatively high accuracy can already be achieved within shorter durations, while performance gradually stabilizes with longer recordings. This result suggests that reliable depression screening can be achieved with shorter recording durations, thereby improving clinical efficiency and practicality.

### 3.2. Feature Analysis

To evaluate the stability and generalizability of our feature selection method, we employed 10-fold cross-validation. In each fold, the two-stage feature selection pipeline was independently executed on the training set, yielding ten distinct feature subsets for analysis.

**Figure 6:**
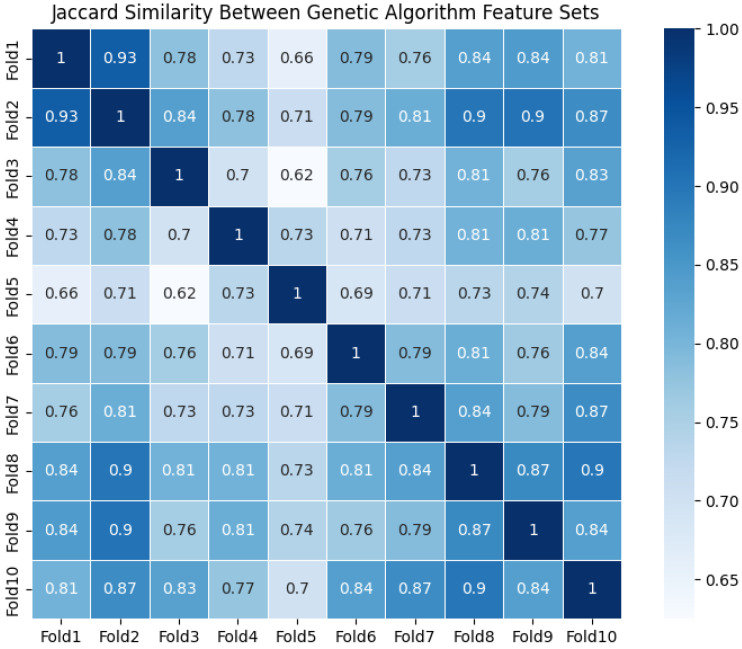
Similarity Coefficients Between Feature Subsets.

As shown in Figure 6, we present the Jaccard similarity heatmap of feature subsets obtained through 10-fold cross-validation to evaluate the stability of our feature selection method. The heatmap reveals high consistency across subsets, with similarity coefficients predominantly ranging between 0.70 and 0.90. Overall, these feature subsets demonstrate strong overlap, indicating that the proposed feature selection pipeline maintains good stability across different data partitions. The average off-diagonal Jaccard similarity coefficient of 0.786 further validates the robustness of our method in repeated cross-validation tests.

**Figure 7:**
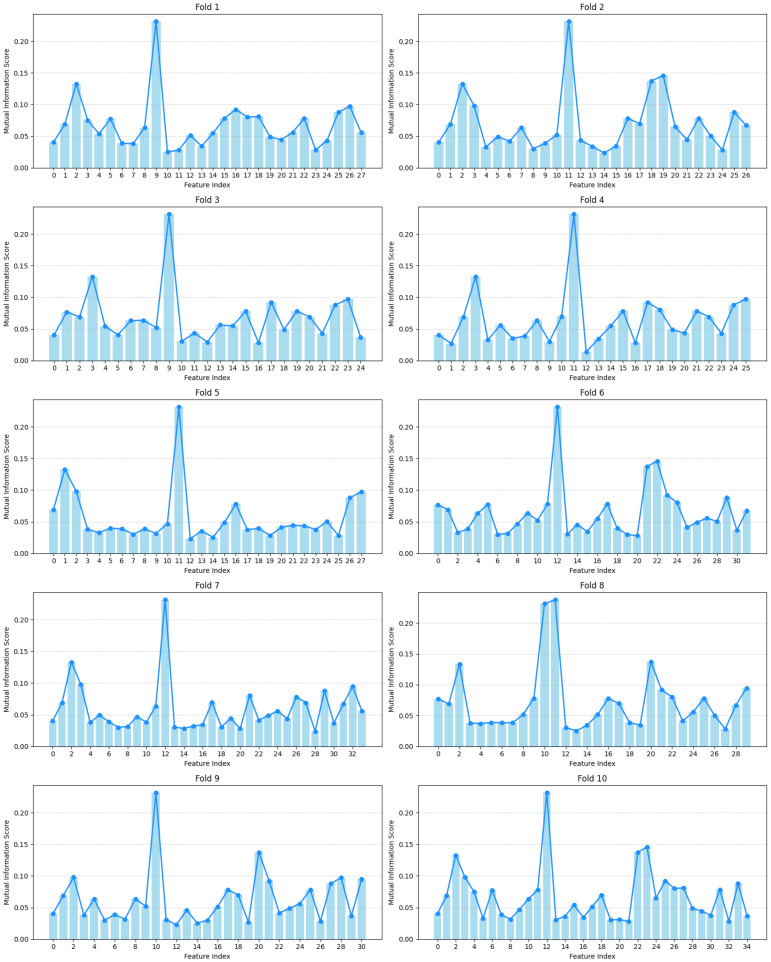
Mutual Information (MI) Scores of Optimal Feature Subsets Across Folds.

As shown in Figure 7, we present the mutual information (MI) scores of features across all subsets obtained from 10-fold cross-validation. By consolidating features from all subsets, we observe consistent MI scores for selected features across different folds. Notably, certain features repeatedly appear in multiple folds with high MI scores, demonstrating their strong relevance to depression classification.

**Figure 8:**
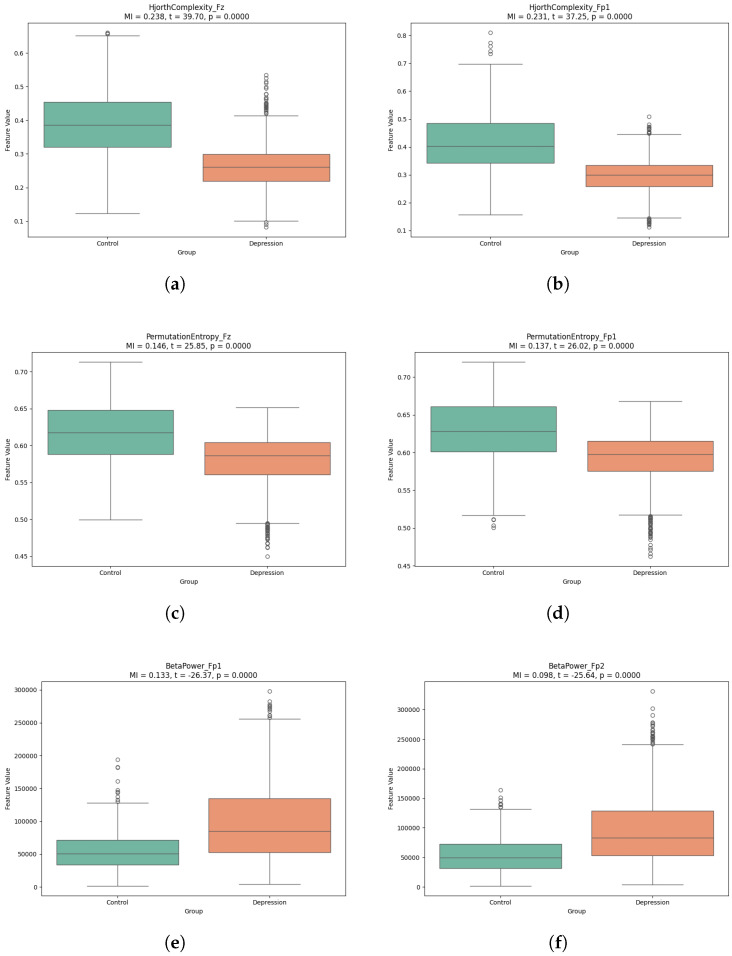
Boxplots of Key Features: (**a**) HjorthComplexity_Fz; (**b**) HjorthComplexity_Fp1; (**c**) PermutationEntropy_Fz; (**d**) PermutationEntropy_Fp1; (**e**) BetaPower_Fp1; (**f**) BetaPower_Fp2.

Notably, to ensure representativeness, we further selected the top six MI-ranked features from this set and conducted significance analysis of their distributions between groups. Figure 8 displays boxplots of these representative features, where points indicate actual observed values (including some outliers) in the samples. These outliers may stem from individual variability or EEG noise, though our analysis focused on the statistical distribution trends across the majority of samples.

As shown in Figure 8a,b, HjorthComplexity_Fz and HjorthComplexity_Fp1 demonstrated high mutual information scores and significant t-values in prefrontal channels, indicating strong discriminative ability between groups through temporal complexity analysis.

As shown in Figure 8c,d, PermutationEntropy_Fz and PermutationEntropy_Fp1 showed similarly high mutual information scores, with significant t-values of 25.848 and 26.022 respectively, validating entropy features’ effectiveness in characterizing EEG signal dynamics.

As shown in Figure 8e,f, BetaPower_Fp1 (MI = 0.133, t = −26.370) and BetaPower_Fp2 (MI = 0.098, t = −25.644) revealed significant between-group differences in beta-band power, suggesting altered neural activity patterns in depression.

### 3.3. Comparison with Other Feature Selection Methods

To validate our method’s effectiveness, we conducted comprehensive comparisons with three classical feature selection approaches using the same dataset. As shown in Table 5, these intra-dataset comparisons provide a fair and rigorous performance evaluation under consistent experimental conditions. For a fair comparison, all feature selection methods were implemented under consistent experimental settings. Specifically, the number of selected features for ACO, LASSO, and PCA was controlled to be comparable with that of the proposed RS+GA framework. In addition, the parameter settings for these methods were determined based on commonly adopted configurations, and all methods were evaluated using the same classifier and cross-validation protocol to ensure reproducibility and fairness. Compared with ACO, LASSO, and PCA, the proposed RS+GA method achieves superior overall performance across multiple evaluation metrics, demonstrating its effectiveness in identifying discriminative feature subsets. The results demonstrate that while GA increases computational cost, RankSearch’s pre-screening reduces feature space by 62%, with overall performance still surpassing metaheuristic algorithms like ACO. Our ranking-guided genetic search achieves an optimal accuracy–efficiency balance, proving particularly suitable for high-dimensional biomedical signal feature selection.

## 4. Discussion

### 4.1. Neurophysiological Basis of Key Features

To further enhance the interpretability of the proposed method, we provide neurophysiological interpretations of the key features identified in this study, including Hjorth Complexity, Permutation Entropy, and Beta Power.

Hjorth Complexity reflects the temporal structure and dynamic variation of EEG signals, indicating the degree of waveform complexity. Previous studies have demonstrated that EEG complexity is closely associated with brain functional states and cognitive processes. In patients with Major Depressive Disorder (MDD), altered complexity patterns have been observed, particularly in frontal brain regions, which may reflect impaired neural adaptability and reduced cognitive flexibility [26,27]. Such alterations suggest a diminished ability of the brain to dynamically respond to internal and external stimuli.

Permutation Entropy is a nonlinear measure that quantifies the irregularity and unpredictability of time-series signals. Reduced entropy values in MDD patients indicate decreased neural signal variability and diminished information processing capacity [28]. Prior studies have shown that entropy-based EEG features are effective biomarkers for depression, as they capture reduced dynamical complexity and altered neural information flow in depressive disorders [29].

Beta band power is closely associated with cortical activation, attention, and emotional regulation [30]. Abnormal beta activity, particularly in prefrontal regions, has been widely reported in individuals with depression. Altered beta power may reflect dysregulation in emotional processing and abnormal neural excitability, which are key characteristics of depressive states [31].

Overall, these findings demonstrate that the selected features are not only statistically discriminative but also neurophysiologically meaningful. This enhances the interpretability of the proposed model and supports its potential clinical applicability. These observations are consistent with previous neurophysiological studies, further supporting the biological plausibility of the proposed feature selection framework.

### 4.2. Comparison with Alternative Methods

As shown in Table 6, we compare our method with existing EEG-based depression detection approaches. The comparison is primarily based on classification accuracy. The proposed method is further evaluated using multiple performance metrics on the HUSM dataset, providing a more comprehensive assessment of its classification performance. It should be noted that these studies are conducted on different EEG datasets with varying experimental protocols, preprocessing strategies, and subject characteristics. Therefore, the results are not directly comparable and should not be interpreted as definitive evidence of the superiority of the proposed method. Instead, Table 6 is intended to provide a supplementary reference context, demonstrating that the proposed method achieves competitive performance within the broader research landscape.

The proposed depression recognition method achieved a competitive overall classification accuracy of 95.08%, with an F1-score of 95.00% and a κ of 90.14%. For feature selection, the two-stage algorithm significantly reduced computational complexity—preliminary feature extraction with mutual information scoring followed by genetic algorithm-based refinement substantially decreased feature dimensionality while maintaining satisfactory classification performance. This process effectively mitigated overfitting risks and enhanced model robustness.

Overall, compared to traditional machine learning methods, our approach demonstrates higher efficiency and accuracy in both feature selection and classification performance, particularly when handling multi-domain features and complex signals, through its integration of features from multiple domains during extraction.

### 4.3. Limitations of the Current Study

The current study has several limitations that require further investigation. First, the public HUSM dataset used in this research remains relatively small in scale, which may impact the classifier model’s generalizability. Although we increased sample quantity through window-sliding augmentation, all samples originated from the same cohort of subjects, potentially introducing individual dependency issues that limit the model’s applicability to broader populations. Future studies could incorporate larger-scale, multi-center datasets covering diverse age groups and cultural backgrounds, or integrate multiple public EEG datasets to enhance the model’s generalizability and practical utility.

Second, the model currently only utilizes three prefrontal electrodes (Fp1, Fz, Fp2) for feature extraction. While these frontal lobe channels have physiological justification for emotion recognition, their limited coverage fails to capture activity from other brain regions. Future work could expand electrode coverage to include more brain areas and incorporate spatial feature extraction techniques like EEG topographic mapping and functional connectivity analysis to enhance the model’s comprehensive characterization of depression-related neural patterns.

In addition, the current study primarily provides feature-level interpretability through feature selection and statistical analysis, while model-level explanations of classifier decisions are not explicitly addressed. Future work could incorporate model interpretability techniques such as SHAP to provide deeper insights into the decision-making process.

Finally, although the RS+GA method combines screening and optimization to improve classification performance, the Genetic Algorithm’s inherent stochastic nature introduces variability. While we assessed stability through multiple runs, subsequent research could implement stability-driven selection strategies such as bootstrap sampling with stability scoring or feature fusion frameworks integrating multiple selection methods to improve consistency and reproducibility.

## 5. Conclusions

This study proposed a novel two-stage feature selection method for depression detection using resting-state EEG signals from Malaysia’s HUSM public dataset. Unlike conventional approaches, our strategy uniquely integrates RankSearch’s efficient preliminary screening with Genetic Algorithm (GA)’s ability to optimize feature interactions, significantly improving classification performance while maintaining computational efficiency. The experiments extracted multi-domain features from three frontal EEG channels (Fp1, Fz, Fp2), constructing a 93-dimensional feature space. RankSearch first eliminated redundant features, while GA refined the subset by exploring feature interactions. The method demonstrated robustness across multiple classifiers (Decision Tree, SVM, XGBoost) and outperformed traditional techniques (ACO, LASSO, PCA) in both accuracy and interpretability through feature importance analysis. Our two-stage framework offers a promising approach for high-dimensional biomedical feature selection, balancing efficiency and effectiveness. The “rapid screening + deep optimization” paradigm can be extended to other physiological signal analyses, demonstrating strong potential for clinical applications requiring both performance and interpretability.

## Figures and Tables

**Table 1:** List of all features.

Function	Features
Time-domain	
Standard deviation, Peak-to-peak amplitude, Root mean square, Hjorth parameters (Activity, Mobility, Complexity)	6
Frequency domain	
Band Power (Delta, Theta, Alpha, Beta, Gamma), Mean Power, Power Spectrum, Peak Frequency, Spectral Asymmetry Index, Band Power Ratios (Alpha/Beta, Theta/Alpha, Theta/Beta)	12
Entropy and Complexity	
Differential Entropy, Sample Entropy, Permutation Entropy, Spectral Entropy, Wavelet Entropy, Fuzzy Entropy, Singular Value Decomposition Entropy, Lempel–Ziv Complexity, Higuchi Fractal Dimension, C0 Complexity, Correlation Dimension, Largest Lyapunov Exponent, Lyapunov Exponent Spectrum	13

**Table 2:** Parameter settings for genetic algorithms.

Parameters	Value
Fitness Function	5-fold CV accuracy using Random Forest
Elitism Strategy	Hall of Fame (keep best 1)
Random Seed Strategy	Dynamically set in each run
Crossover Probability	0.8
Gene Mutation Probability	0.05
Population Size	50
Number of Generations	20

**Table 3:** Performance Metrics Comparison Across Classifiers.

Model	Acc	Sen	Spec	F1	κ	AUC
DT	0.9237	0.9270	0.9209	0.9300	0.8469	0.9239
KNN	0.9458	0.9661	0.9254	0.9457	0.8915	0.9458
RF	0.9441	0.9599	0.9304	**0.9500**	0.8879	0.9451
SVM	0.9458	**0.9672**	0.9272	**0.9500**	0.8913	0.9472
XGBoost	**0.9508**	0.9599	**0.9430**	**0.9500**	**0.9014**	**0.9514**

Note: Bold indicates the best performance among all classifiers.

**Table 4:** Performance Comparison of Different Feature Sets.

Method	Average					
	Acc	Sen	Spec	F1	*κ*	AUC
93 features	0.9546	0.9618	0.9483	0.9576	0.9089	0.9550
73 features	0.9580	0.9669	0.9502	0.9579	0.9157	0.9586
30 features	0.9420	0.9560	0.9294	0.9451	0.8838	0.9427
10 features	0.8532	0.8505	0.8560	0.8609	0.7057	0.8533

**Table 5:** Classification Performance Comparison of Different Feature Selection Methods.

Methods	Acc	Sen	Spec	F1	κ	AUC
ACO	0.8665	0.8681	0.8647	0.8666	0.7329	0.8664
LASSO	0.5211	0.4491	0.5929	0.5480	0.0419	0.5210
PCA	0.8732	0.8720	0.8566	0.8517	0.7445	0.8533
This Work	**0.9420**	**0.9560**	**0.9294**	**0.9451**	**0.8838**	**0.9427**

Note: Bold indicates the best performance among Different Feature Selection Methods.

**Table 6:** Comparison with Existing Models.

Authors	FS_Model	Classifier	Acc
Erguzel [13]	IACO	SVM	80.19%
Hassan [32]	EN, MI, $\chi^{2}$ test, FFS-SGD, SVM-RFE, mRMR	LDA, SVM, RF, GBDT	93.54%
Bhadra [33]	RFE, MI, PSO, GA, FA	SVM, LR, DT, RF, GB	88.46%
Li [34]	BF, GSW, LFS, RS	BN, SVM, RF, LR, KNN	92.00%
Cai [11]	WSE, CAE, PCA, GRAE	SVM, KNN, DT, LR, RF	76.40%
This Work	RS+GA	DT, KNN, RF, SVM, XGBoost	**95.08%**

Note: Bold indicates the best performance among different models.

## Data Availability

The original data presented in the study are openly available from FigShare at https://figshare.com/articles/dataset/EEG_Data_New/4244171 (accessed on 27 March 2026).
